# Missense Mutation in *CAPN1* Is Associated with Spinocerebellar Ataxia in the Parson Russell Terrier Dog Breed

**DOI:** 10.1371/journal.pone.0064627

**Published:** 2013-05-31

**Authors:** Oliver P. Forman, Luisa De Risio, Cathryn S. Mellersh

**Affiliations:** 1 Kennel Club Genetics Centre, Animal Health Trust, Kentford, Newmarket, Suffolk, United Kingdom; 2 Neurology/Neurosurgery Service, Centre for Small Animal Studies, Animal Health Trust, Kentford, Newmarket, Suffolk, United Kingdom; University of Florida, United States of America

## Abstract

Spinocerebellar ataxia (SCA) in the Parson Russell Terrier (PRT) dog breed is a disease of progressive incoordination of gait and loss of balance. Clinical signs usually become notable between 6 and 12 months of age with affected dogs presenting with symmetric spinocerebellar ataxia particularly evident in the pelvic limbs. The degree of truncal ataxia, pelvic limb hypermetria and impaired balance is progressive, particularly during the initial months of disease. A certain degree of stabilisation as well as intermittent worsening may occur. At the later stages of the disease ambulation often becomes difficult, with owners often electing to euthanise affected dogs on welfare grounds. Using a GWAS approach and target-enriched massively-parallel sequencing, a strongly associated non-synonymous SNP in the *CAPN1* gene, encoding the calcium dependent cysteine protease calpain1 (mu-calpain), was identified. The SNP is a missense mutation causing a cysteine to tyrosine substitution at residue 115 of the *CAPN1* protein. Cysteine 115 is a highly conserved residue and forms a key part of a catalytic triad of amino acids that are crucial to the enzymatic activity of cysteine proteases. The *CAPN1* gene shows high levels of expression in the brain and nervous system and roles for the protein in both neuronal necrosis and maintenance have been suggested. Given the functional implications and high level of conservation observed across species, the *CAPN1* variant represents a provocative candidate for the cause of SCA in the PRT and a novel potential cause of ataxia in humans.

## Introduction

Spinocerebellar ataxia (SCA) also referred to as hereditary ataxia has been reported in several related fox terrier breeds including the Smooth-Haired Fox Terrier (SHFT) [1], [2], [3], the Jack Russell Terrier (JRT) [4], [5], [6] and the Parson Russell Terrier (PRT) [4]. The JRT is similar, although shorter legged than the PRT, but is not a registered ‘pure breed’ with the UK Kennel Club.

Clinical signs of SCA are usually recognised by the owners when the dogs are 2 to 9 months of age [4], [6]. Initially the dogs’ owners may notice incoordination, pelvic limb swaying when walking, and difficulty in climbing stairs and jumping. As the disease progresses, a characteristic ‘prancing’ or ‘dancing’ type of gait is observed, especially affecting the pelvic limbs. Severely affected animals frequently fall and have difficulty returning to a standing position. Neurological examination reveals symmetric spinocerebellar ataxia, characterised by hypermetria and spasticity particularly in the pelvic limbs. Postural reactions may be delayed and hypermetric. Spinal reflexes are normal to increased and cranial nerve examination is generally unremarkable [4], [6]. In one study, haematology, serum biochemistry, urinalysis, cerebrospinal fluid analysis, radiography, myelography, and spinal computed tomography did not identify any abnormalities in affected PRTs and JRTs [4]. Brain stem auditory-evoked potentials (BAEPs) revealed abnormalities in some, but not all, of the affected dogs [4]. The BAEP abnormalities were characterised by absence of waves III, IV and V. Progression of neurological dysfunction is variable. Generally clinical deterioration results in severe difficulty ambulating, with owners often electing to euthanise affected dogs on welfare grounds. However, one dog with a milder disease course survived for 15 years [4].

Post-mortem examination shows no macroscopic abnormalities of the central nervous system. Histopathological examination reveals bilateral symmetrical myelopathy, predominantly an axonopathy combined with myelin loss, in the dorsal and ventral or ventromedial funiculi. Swelling of axons and dilatation of myelin sheaths with loss of myelin adjacent to a mild astrogliosis were observed primarily in the spinocerebellar tracts of the cervical cord but were also observed in all parts of the brain in the PRT and in the JRT [4], [6]. Moderate diffuse gliosis, marked loss of myelinated nerve fibers, and argyrophilic axonal spheroids were detected in central auditory pathways, including superior olivary nuclei, cochlear nuclei, connecting nerve fibers between these nuclei and the trapezoid body, and the lateral lemniscus. These lesions correlate with the abnormalities reported on BAEPs as the neural generators of wave III and IV are the trapezoid body and lateral lemniscus, respectively. The SHFT was initially reported to have spinal cord involvement only [2], [3], however, a more recent study reported histopathological evidence for degenerative changes in the brainstem and clinical evidence of brain involvement [1].

An autosomal recessive mode of inheritance was suspected in the Smooth-Haired Fox Terriers [2], [3]. In the study including PRTs and JRTs, complex segregation analysis across three families containing 115 individuals suggested a hereditary cause for the ataxia with a polygenic model most likely, although a major gene effect with additional polygenic factors was not excluded [4]. Hereditary ataxia has been reported also in JRTs with concurrent other neurological disorders including behavioural changes, seizures, respiratory distress (Wessmann 2004), myokymia and neuromyotonia [5], [7] suggesting phenotypic and genetic heterogeneity.

Genetic mechanisms for three types of inherited canine cerebellar disorders have been described in the literature to date. Retrotransposon insertion within the gene encoding glutamate receptor, metabotropic 1 (*GRM1*) has been associated with neonatal cerebellar ataxia in the Coton de Tulear dog [8], [9]. Cerebellar ataxia in the Finnish Hound was shown to be caused by a missense mutation in the sel-1 suppressor of lin-12-like (*SEL1L*) gene [10]. Most recently neonatal cerebellar cortical degeneration in the Beagle was associated with an 8 bp deletion in the gene encoding beta-III spectrin (*SPTBN2*), which is known to caused spinocerebellar type 15 in humans [11]. SCA in the PRT has a later onset and slower progression rate in comparison to the early-onset canine cerebellar ataxias with known molecular mechanisms and is therefore likely to have a novel genetic cause. To improve our understanding of SCA in the PRT, we collected a set of SCA cases and controls to perform a genome-wide association study (GWAS) to elucidate the mode of inheritance and identify the causal mutation(s). Results of the GWAS and identification of a strongly associated and highly provocative potential causal mutation are described.

## Results

**Figure 1:**
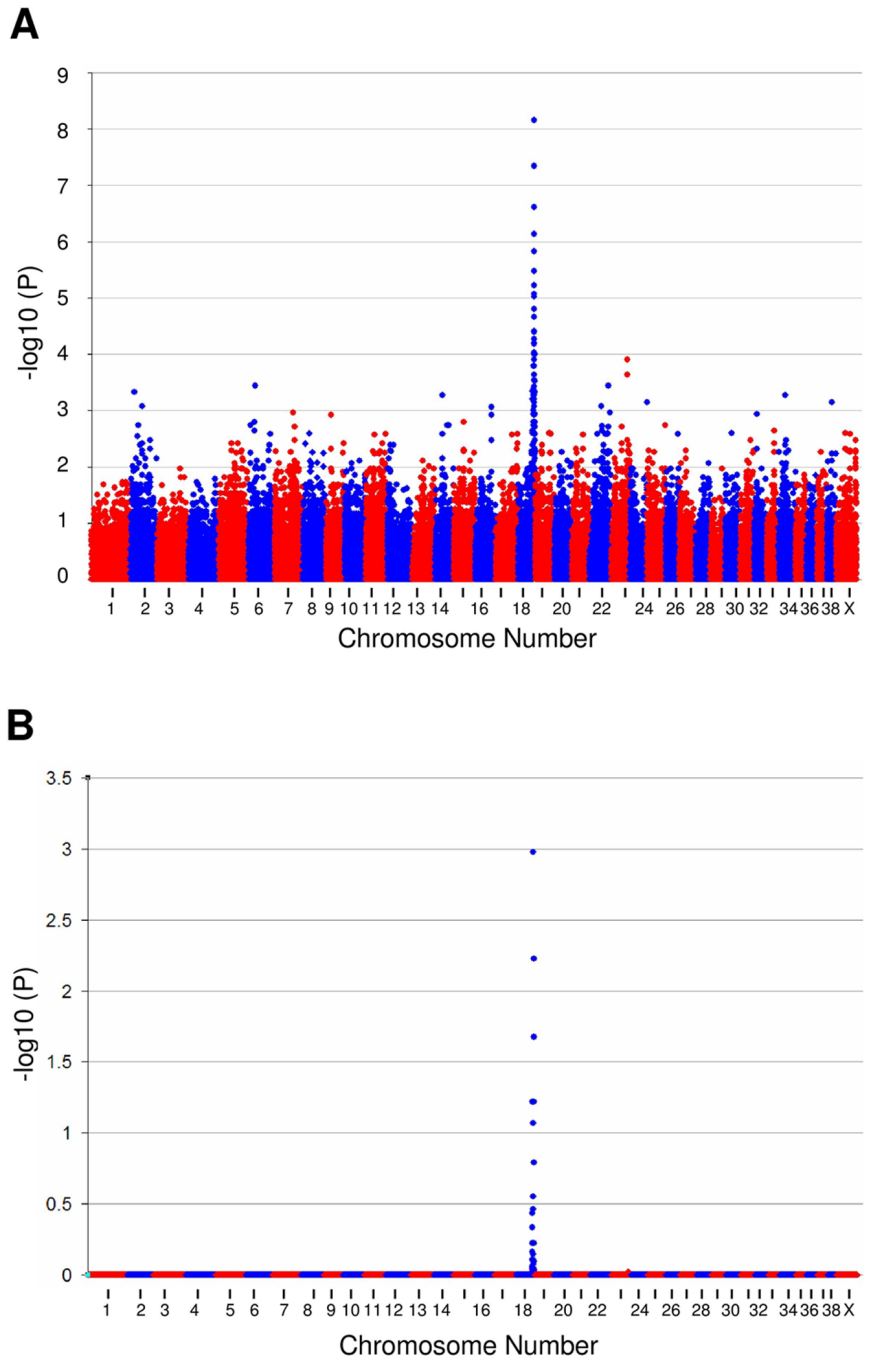
Allelic association analysis plots for SCA in the PRT. Allelic association analysis on 16 SCA cases and 16 controls. Each dot represents a single SNP, with -log10 (p) values on the y-axis plotted against genome position (split into chromosomes) on the x-axis. (A) Raw unadjusted log(p) values with a strong statistical signal indicated on chromosome 18 (P_raw_ = 7.04×10^−9^). (B) Plot of -log10(p) values after 100,000 maxT permutations analysis to correct for multiple testing, showing a single peak reaching genome-wide significance on chromosome 18 (P_genome_ = 1.06×10^−3^).

A GWAS was performed using 16 SCA cases and 16 controls. DNA samples were all successfully genotyped on the Illumina CanineHD array achieving call rates of >99.8%. Allelic association analysis was performed using the statistical analysis package PLINK which was implemented at the Linux command prompt. After exclusion of SNPs with a minor allele frequency of less than 0.05 and genotyping success rate of less than 0.95, 126,225 SNPs remained. The genomic inflation factor based on the median chi-squared value was 0.8 indicating no stratification between the case and control populations, which was further confirmed by multidimensional-scaling (MDS) and quantile-quantile (QQ) plotting (Figure S1). The genomic inflation value of <1 is most likely because of the use of closely related case-control pairs in the GWAS (two sibling pairs, six half-sibling pairs and four offspring-parent pairs included - Table S1). Basic allelic association analysis on the filtered SNP set revealed a strong statistical signal on chromosome 18 (P_raw_ = 7.04×10^−9^) (Figure 1A). To correct for multiple testing, allelic association analysis was performed using 100,000 MaxT permutations in PLINK. A single signal on chromosome 18 of genome-wide significance remained (P_genome_ = 1.06×10^−3^ ) (Figure 1B). Correction for population substructure and relatedness was performed using a mixed model, implemented in the statistical package R. The single strong statistical signal remained (P_corrected_ = 9.89×10^−10^) (Figure S2). Results were suggestive of a simple autosomal recessive mode of inheritance for the disorder.

**Figure 2:**
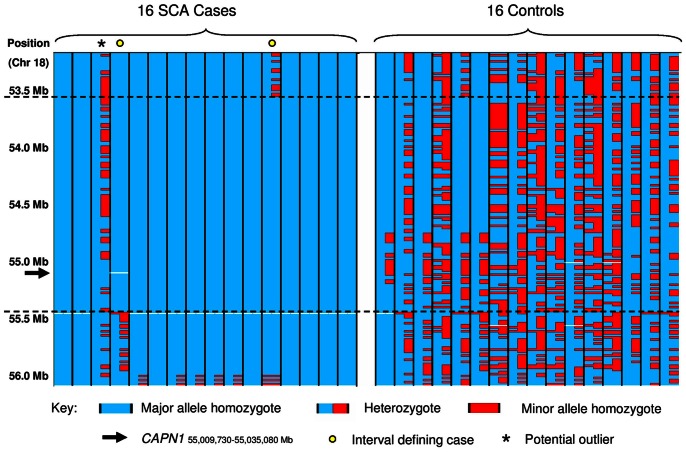
Raw genotyping data across the disease associated region for SCA. Graphical overview of the raw genotyping data across the SCA critical region. Each column represents one individual, and SNP markers are listed in the rows with the approximate position indicated. Major alleles are highlighted in blue and minor alleles in red. Interval defining cases are marked by a circle at the top of individual columns. The outlying case which does not share the disease associated haplotype across the interval is marked with an asterisk.

Genotyping data from the region of genome-wide significance on chromosome 18 revealed that all but one of the 16 cases were homozygous for a shared haplotype (Figure 2). Recombination events in two individuals defined the disease-associated interval as chr18∶53,533,360–55,418,743 based on the CanFam2 genome build. The disease-associated region for SCA contained 91 genes and was syntenic to chromosome 11 of the human genome (Figure S3). Genes within the interval were assessed for potential involvement in SCA. The gene encoding beta-III spectrin (*SPTBN2*) was a particularly strong candidate, as mutations in the gene have been shown to cause spinocerebellar ataxia type 5 (SCA5) in humans [12] as well as an inherited neurological disorder in the dog [11]. Exon resequencing of the gene revealed no potentially causal coding polymorphisms.

The entire disease-associated interval was subsequently interrogated using a target-enriched massively parallel sequencing approach. Sequencing on a single lane of the Illumina HiSeq2000, produced a 19.64 Gb dataset of 51 bp paired-end reads, with 79% of bases in the region achieving 10x read coverage. In total 7,024 SNPs and 1,507 indels were identified across the disease-associated interval, with 541 variants segregating in accordance with disease status. Sequence data from the same region, generated from an additional 25 control individuals of the same/different breed, allowed 399 SNPs to be excluded from consideration, reducing the number of segregating SNPs to 142 (Table S2). Segregating variants were considered by assessing conservation levels and variant effect predictions. Two SNPs were considered as potentially causal. These were a missense mutation (C115Y) in the gene encoding the large subunit of calcium dependent cysteine protease, μ-calpain (*CAPN1*) and a missense mutation (E24K) in the vacuolar protein sorting 51 homolog (*VPS51*). The two candidate SNPs were investigated by genotyping an additional cohort of 227 PRTs consisting of 27 cases and 200 control individuals. Results are summarised in Table 1. As four control individuals were homozygous for the disease associated *VPS51* SNP allele, the variant could be excluded as potentially causal.

**Table 1:** Results summary of 227 PRTs genotyped for the *CAPN1* and *VPS51* disease-associated SNPs.

	CAPN1 SNP		VPS51 SNP	
Genotype	Case	Control	Case	Control
**w/t homozygous**	3	133	3	128
**Heterozygous**	1	67	1	68
**Mutant homozygous**	23	0	23	4

Additionally a multibreed panel consisting of 96 healthy individuals representing 32 dog breeds was genotyped for both the *VPS51* and *CAPN1* SNP variants. The non-reference *VPS51* allele was found in four individuals (one Tibetan Spaniel, one Miniature Poodle, and two Doberman Pinschers), providing further evidence that the *VPS51* variant was likely to be a common polymorphism. All 96 individuals were homozygous for the wild-type/reference *CAPN1* allele.

**Figure 3:**
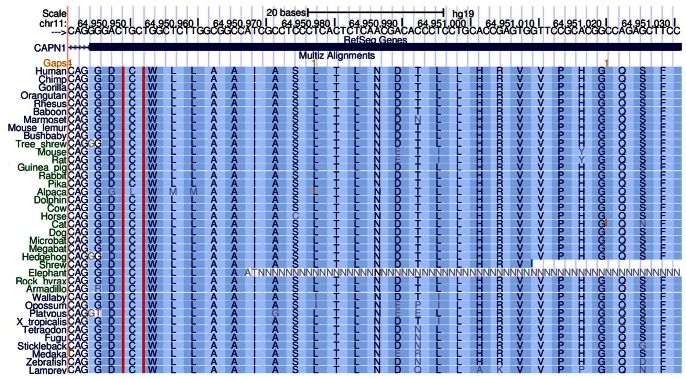
Multispecies alignment across residue 115 of the calpain peptide. Alignment across 46 vertebrate species visualised using the UCSC genome browser (human genome build hg19) to assess the level of cross-species conservation for the *CAPN1* gene.

Multispecies alignments revealed that cysteine residue 115 of *CAPN1* is highly conserved (Figure 3). Of the 38 aligned species, all had a cysteine residue at the 115 position, apart from alpaca which has a leucine residue. A 1 bp insertion at residue 124 puts the alpaca transcript out of frame however, possibly suggesting that the gene is non-functional in this species.

To help assess whether the *CAPN1* amino acid change could have an important functional effect, three computational methods of predicting mutation effects were used - SIFT (Sorting Intolerant from Tolerant), Polyphen (Polymorphism Phenotyping) and Mutation Taster [13], [14], [15]. All three methods suggested the mutation to be damaging or disease causing, with high confidence level values, with the Mutation Taster predicting loss of a catalytic domain.

Genome-wide messenger RNA sequencing (mRNA-seq) of one SCA case and one control cerebellum tissue sample was used to assess gene expression levels across the disease-associated interval. On visual inspection of read alignments expression levels for the disease-associated region appeared to be equivalent for the case and control across the disease-associated region, enabling a change in gene expression to be excluded as the cause of SCA.

Five SCA affected JRTs were genotyped for the *CAPN1* SNP. Of the five dogs three were homozygous wild-type, one was heterozygous and one was homozygous for the disease-associated allele. This may suggest that the identified locus is not a major cause of ataxia in the JRT breed. All eight outliers which were reported as clinically affected, but were not homozygous for the *CAPN1* mutation were further investigated by genotyping 48 informative SNP markers distributed across the disease-associated interval. Results showed that none of the outliers were homozygous for any part of the disease-associated interval, which in turn suggested a different clinical or genetic cause for the clinical signs in these cases, rather than the possibility that the *CAPN1* mutation was not causal. Genotyping results are summarised in Figure S4.

## Discussion

**Figure 4:**
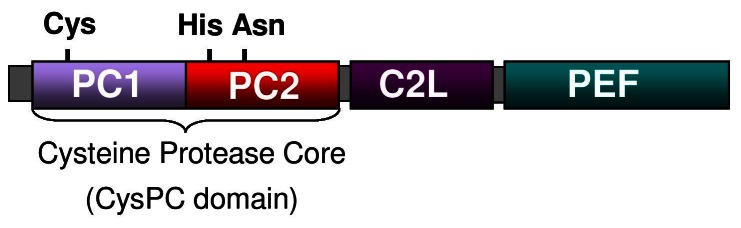
The schematic structure of calpain. Classical calpains, which include CAPN1, 2, 3b, 8, 9 11, 12, 13, 14, consists of two protease core domains (PC1 and PC2), a C2 like domain (C2L) and a penta EF-hand domain (PEF). The PC1 and PC2 make up the cysteine protease core. All calpains have a protease core domain, but the other domains differ in arrangement for the non-classical calpains.

In this investigation we have identified a *CAPN1* mutation that is strongly associated with SCA in the PRT, using a GWAS approach followed by target-enriched massively parallel sequencing of the disease-associated interval. Mutations in *CAPN1* have not previously been associated with ataxia in any species. The *CAPN1* gene encodes an intracellular calcium dependent cysteine protease, which is a member of the calpain family and papain superfamily of cysteine proteases found throughout the plant, animal and fungal kingdoms [16], [17]. Conventional calpains consist of an N-terminal anchor helix region, two protease core domains (PC1 and PC2), a C2 like domain (C2L) and a penta EF-hand calcium binding domain (PEF) (Figure 4). The *CAPN1* protein forms heterodimeric structures with the small regulatory subunit *CAPNS1*, interacting with the fifth EF-hand motif. On binding of calcium, conformational changes result in formation of a catalytic triad of cysteine, histidine and asparagine residues and activation of the enzyme [18].

The disease-associated *CAPN1* mutation is a non-synonymous G to an A base substitution at position 344 of the *CAPN1* transcript, resulting in substitution of a cytosine residue for a tyrosine (C115Y). This 115 cysteine residue corresponds to the catalytic cysteine residue that forms part of a catalytic triad with histidine and asparagine, and is therefore critical to the enzymatic properties of the protein (http://www.uniprot.org/uniprot/P07384). Consequently, substitution of this residue is likely to have a detrimental effect on enzyme activity, and could potentially result in loss of function. An extremely high level of conservation was observed across species for *CAPN1* orthologues at the amino acid level for the 115 cysteine residue. High levels of conservation within species for calpain family members (paralogues of *CAPN1*) further suggest the critical importance of the residue. Predictive tools suggest the mutation to be potentially pathogenic. Collectively the evidence presents a strong case for the *CAPN1* mutation to be deleterious.

Calpains are well associated with nervous function. Calpains have been associated with neuronal necrosis, with proteolytic activity increasing as cellular calcium levels rise due to loss of homeostasis after trauma [19]. Experimentally induced brain trauma by ischaemia (narrowing of the blood vessels) was shown to result in increased proteolysis of the calpain substrate frodin (alpha spectrin), a major cytoskeletal protein, an event hypothesised to be part of a cascade leading to neuronal cell death [20]. Calpain inhibitors have therefore been suggested as potential therapeutics for traumatic brain injury [21], [22]. Calpains have also been linked to roles in long term potentiation and Alzheimer’s disease [23]. Despite links to neuronal death and disease, there is evidence that calpains may actually contribute to dendrite remodelling after neural injury, suggesting a maintenance role [24].

Advances in the understanding of calpains have been made by analysing targeted gene knockouts. The importance of the ubiquitously expressed μ/m calpains (*CAPN1* and *CAPN2*) was shown by targeted knockout of the gene encoding the small regulatory subunit CAPNS1 (*CAPN4*), which resulted in embryonic lethality [25]. Targeted deletion of mouse *CAPN2* also resulted in embryonic lethality, but *CAPN1* null mice appear phenotypically normal, apart from an observed reduction in platelet aggregation, although no effects on bleeding time were seen [26], [27]. Although genomic comparisons and predictive tools strongly suggest that the *CAPN1* mutation is likely to be pathogenic, this contradicts the clinically normal phenotype observed in the *CAPN1* null mouse. Conversely, there have been no suggestions of platelet disorders in dogs homozygous for the *CAPN1* variant, although this potential defect has not been formally investigated. One possibility is that null mice do not have significant longevity or are euthanised before manifestation of clinical signs. Another possibility is that the *CAPN1* gene has a slightly differing role in the mouse with the calpain family having a level of redundancy, allowing other family members to compensate for loss of calpain 1 activity. A third possibility is that the *CAPN1* mutation is not the cause of the SCA but is in fact a marker in linkage disequilibrium with the disorder, although the exact residue changed by the *CAPN1* variant is highly provocative.

A possible role for calpain 1 in neuronal maintenance and remodelling role best fits the potential for a defective calpain 1 protein being the cause of SCA in the PRT. SCA is largely a disease of motor neurone degeneration in the spinocerebellar tract, with Wallerian type degeneration observed on histopathological examination. Defective maintenance mechanisms due to lack of calpain 1 leading to neurite degeneration and necrosis would explain these observations, although processing of substrates by calpain 1 have been implicated in neurite degeneration confusing calpain’s role and potentially suggesting involvement in multiple molecular processes and pathways [28], [29].

The gene encoding beta-III spectrin (*SPTBN2*) provided a strong candidate gene within the disease associated region. Mutations in *SPTBN2* have been shown to cause SCA5 in humans [12] and recently neonatal cerebellar cortical degeneration in the Beagle dog [30]. Beta-III spectrin is primarily expressed in the nervous system and the highest levels of expression are found in Purkinje cell soma and dendrites [31]. Beta-III spectrin has been shown to stabilise the glutamate transporter *EAAT4* at the plasma membrane of the Purkinje cells [32], facilitate protein trafficking by linking the microtubule motor to vesicle-bound cargo [33] and maintain a high density of sodium channels within the soma and dendrites of Purkinje cells [34]. Beta-III spectrin is critical for development of Purkinje cells [35]. On exon resequencing of *SPTBN2* no non-synonymous SNPs, frameshift or splice site variants were identified excluding coding changes as a potential cause of SCA in the PRT. Targeted resequencing of the SCA disease-associated region enabled both coding and non-coding regions of the *SPTBN2* to be investigated. No potentially causal variants were identified. Data from mRNA-seq experiments provided further evidence regarding the *SPTBN2* transcript and enabled mis-splicing and changes in expression levels to be excluded as potential causes of SCA. The *SPTBN2* gene was therefore excluded from further consideration.

Although similar clinical signs of ataxia are shared in both SCA and SCA5, other clinical features suggest potentially different disease mechanisms. Clinical features of SCA5 suggest a predominately cerebellar disease [36], whereas histopathological examination of SCA cases suggested limited cerebellum pathology, with degeneration of the brain stem and spinocerebellar tract involved in disease progression. This evidence suggests a different genetic cause for SCA and SCA5, although it is possible for different mutations in the same gene to result in variable phenotypes.

Four PRT and four JRT which displayed clinical signs of SCA, but were not homozygous for the *CAPN1* disease-associated allele were identified in the study. Efforts were made to follow up these discordant cases by contacting owners and veterinarians. Seven of the cases had clinical signs consistent with SCA, although full neurological examinations had not been performed to rule out other causes of spinocerebellar disease. On review by a veterinary neurologist (LDR) one outlying case (the outlier from the GWAS) was deemed not to fit the case definition because typical pelvic limb hypermetric and spastic gait with truncal ataxia was not reported. Of the eight discordant cases, six were homozygous for the wild-type *CAPN1* allele and two were heterozygous. Given the available information it is impossible to rule out a second genetic cause of a clinically similar form of hereditary ataxia in the PRT and JRT. Anecdotal evidence suggests a neonatal onset form of ataxia also segregates within the JRT and PRT dog breeds.

### Conclusions

Using a GWAS approach and target-enriched massively parallel sequencing a disease-associated SNP in *CAPN1* has been identified. The SNP is a missense mutation causing a cysteine to tyrosine substitution at residue 115 of the calpain protein. Cysteine 115 is a highly conserved residue and forms a key part of a catalytic triad of amino acids that are crucial to the enzymatic activity of cysteine proteases. Given the function and high level of conservation, substitution of the cysteine residue is highly likely to have a negative effect on the activity of the enzyme, although functional studies would be required to provide formal evidence to support this hypothesis and to assess the extent of activity loss. Loss of *CAPN1* activity as a cause of SCA is difficult to prove, although a suggested role for *CAPN1* in neuronal maintenance fits with the pathogenesis of the disease based on histopathological evidence. The finding represents the first association of a mutation in *CAPN1* with spinocerebellar ataxia and may represent a novel candidate gene for ataxia in human patients.

## Materials and Methods

### Ethics Statement

Cerebellum tissue samples were collected post-mortem after dogs had been euthanised on welfare grounds. Euthanasia was carried out solely to alleviate suffering and no healthy individuals were sacrificed for use in this study. Euthanasia was carried out in accordance with the Veterinary Surgeons Act 1966 and under the auspices of the RCVS. DNA was collected by buccal swabbing, which is a relatively non-invasive procedure that does not require a license. All samples used in this study were collected after permission had been granted by PRT dog owners. This study did not require ethics committee approval as euthanasia was carried out on welfare grounds only and DNA was collected using a relatively non-invasive procedure which did not require a license.

### Sample Collection and Nucleic Acid Extraction

All DNA samples were collected from privately owned pet dogs by buccal swabbing and extracted using the QIAamp midi DNA extraction kit (Qiagen). Cases were owner reported with video evidence provided for four individuals. Case details of individuals included in the GWAS stage were reviewed by a veterinary neurologist (LDR) (Table S1). To provide a better description of the phenotype, LDR (a specialist in veterinary neurology) performed a neurological examination and measured BAEPs in one of the affected dogs which was homozygous for the *CAPN1* mutation. Video footage and clinic-diagnostic details of this dog have been provided (Video S1). The neurological examination of this PRT revealed the typical gait and neurological deficits of SCA and the BAEP revealed absence of waves III and IV, suggestive of dysfunction of the the trapezoid body and lateral lemniscus, respectively, as previously described in PRT with hereditary ataxia (Figure 5 in Wessmann 2004) [4]. Controls were dogs that were reported as clinically healthy with no signs of ataxia at or above 4 years of age. The additional panel of 96 dogs from 32 other breeds genotyped for both the *CAPN1* and *VPS51* SNPs were all reported by their owners to be healthy and had been recruited to participate in other unrelated studies. Cerebellum tissue samples were collected post-mortem and total RNA extracted using the RNeasy midi kit (Qiagen).

### GWAS Analysis

DNA samples from 16 PRT cases and 16 PRT controls were genotyped using the Illumina CanineHD SNP genotyping array that comprises 173,662 SNPs. The SNP genotyping dataset was analysed for association using the statistical package PLINK [37]. Sample call rates for all individuals were >99%. SNPs with a genotyping call rate of <95% and/or minor allele frequency of <5% were discarded. The strongest statistical signal from the unadjusted association analysis was termed P_raw_. Correction for multiple testing was performed using 100,000 MaxT permutations in PLINK. The strongest statistical signal after permutations was termed P_genome_. Correction for genomic inflation was performed using a mixed model approach implemented in the statistical package R [38]. The strongest statistical signal after correction was termed P_corrected._

### *SPTBN2* Resequencing

The *SPTBN2* gene was exon resequenced using Sanger sequencing methodology. Templates for sequencing were created by PCR. Reaction mixes consisted of 1.5 mM dNTPs, 1×PCR buffer, 0.8 µM forward and reverse primer, 1.2 U HotStarTaq polymerase (Qiagen), 2 µl genomic DNA and ultrapure water to give a final volume of 12 µl. Sequencing was performed using BigDye v3.1 terminator chemistry (Applied Biosystems) for capillary electrophoresis on ABI3130×l genetic analysers. PCR primers are listed in Table S3.

### Target-enriched Massively-parallel Sequencing

Libraries were created for sequencing on the Illumina platform using the SureSelectXT solution based target enrichment library preparation kit (Agilent Technologies). RNA bait probes (120 bp) were designed to give 2x probe coverage of target regions using the online tool e-array (https://earray.chem.agilent.com/earray/). The total number of baits designed was 32,220, covering 2.21 Mb of the 3.84 Mb target region after repeat masking (56.7% base coverage). Genomic DNA (5 µg) from two SCA cases, and three control dogs was used to prepare libraries for sequencing. DNA was Covaris fragmented at the Eastern Sequence and Informatics Hub, Cambridge, UK, followed by in-house library preparation using the SureSelectXT library preparation kit. Paired-end sequencing (51 bp reads) was carried out on a single lane of an Illumina HiSeq2000 at the Wellcome Trust Centre for Human Genetics, University of Oxford, UK, producing a 19.64 Gb dataset. The sequencing dataset was submitted to the Sequence Reads Archive (SRA), accession number SRP018940.

### Sequencing Data Analysis

Reads were aligned to the canine reference genome (CanFam2) using BWA [39]. SNP and indel calls were made using GATK [40]. Aligned reads were viewed using The Integrative Genomics Viewer (IGV) [41]. Polymorphisms occurring in exonic regions causing non-synonymous changes and in splice donor or acceptor sites were considered as candidate mutations. Candidate mutations were considered potentially causal if they were homozygous mutant in cases and either heterozygous or homozygous wild-type in controls.

### mRNA-seq

Libraries were prepared using the NEBNext mRNA Sample Prep Master Mix Set 1, consisting of RNA fragmentation, first strand cDNA synthesis, second strand synthesis, end repair, dA tailing, and PCR amplification modules. Reverse transcription of RNA fragments was performed using Superscript II Reverse Transcriptase (Life Technologies). Clean-up after each module was performed using the RNeasy mini or QIAquick PCR purification mini kit (Qiagen). The adaptor ligated library was size selected by band excision after agarose gel electrophoresis, and purified using the QIAquick gel extraction kit (Qiagen) before PCR amplification, using primers for Illumina paired-end multiplexed sequencing. The final mRNA-seq library was quantified by qPCR using the Kapa library quantification kit (Kapa Biosystems). Libraries were sequenced at the Wellcome Trust Centre for Human Genetics, Oxford, UK, producing a 3.31 Gb dataset for the SCA case and a 3.34 Gb dataset for the control. Aligned reads were viewed using The Integrative Genomics Viewer (IGV). The sequencing dataset was submitted to the Sequence Reads Archive (SRA), accession number SRP012049.

### Genotyping of the *CAPN1* and *VPS51* Disease-associated SNP Variants

All SNPs were genotyped using allelic discrimination methodology (TaqMan - Life Technologies). Reactions were carried out in 8 µl volumes consisting of 4 µl Kapa probe fast (Kapa Biosystems), 0.2 µl 40x probe mix, 2 µl genomic DNA and 1.8 µl ultrapure water. Cycling parameters were 40 cycles of 95°C for 3 seconds and 60°C for 15 seconds. Primer and probe sequences are listed in Table S3.

### Genotyping-by-sequencing

A genotyping-by-sequencing method was used to genotype 48 SNP markers. PCRs contained 0.2 mM dNTPs, 1×Qiagen reaction buffer, 2.5 mM MgCl_2_, 2 µl genomic DNA, 0.3 µl Qiagen HotStarTaq, 0.2 µM of each primer (96 in total) and molecular grade water to a total volume of 17 µl. Cycling parameters were 95°C for 5 minutes, 25 cycles of 95°C for 30 seconds, 60°C for 2 minutes, and a final elongation at 60°C for 15 minutes. The Illumina sequencing adaptor was directly ligated onto the A’ tail created by PCR using T4 ligase (NEB). Index sequences were incorporated during a library amplification stage and paired-end 100 bp sequencing was carried out on an Illumina Miseq. Data was aligned to the CanFam3 genome build on the Miseq, and data analysed by visualising in IGV.

## Supporting Information

Figure S1 **QQ and MDS plots for the GWAS dataset.**
(TIF)

Figure S2 **Allelic association analysis plot after correction for population stratification using a mixed model approach.**
(PDF)

Figure S3 **SCA disease-associated interval and human syntenic chromosome.**
(TIF)

Figure S4 **Genotyping results of the eight outlier cases for 48 SNPs across the disease-associated interval.** The outlier cases are not homozygous for the disease-associated haplotype.
(PDF)

Table S1 **Summary of cases used in the GWAS.**
(PDF)

Table S2 **Summary of the 142 SNPs and indels that segregated with disease status.**
(PDF)

Table S3 **List of primer names and sequences used in the study.**
(DOC)

Video S1 **Parson Russell Terrier with SCA.** 6 year, 4 month old, male neutered PRT with SCA. Note the characteristic prancing or dancing type of gait, especially affecting the pelvic limbs. There is truncal ataxia, impaired balance and pelvic limb hypermetria and spasticity. The owner of this PRT detected initial signs of SCA when the dog was approximately 10 months old. Progressive deterioration with periods of stabilisation has occurred. Neurological examination and BAEPs were consistent with those previously reported in PRTs and JRTs with hereditary ataxia.
(WMV)
