# Supplementary figures and images for: Missense Mutation in CAPN1 Is Associated with Spinocerebellar Ataxia in the Parson Russell Terrier Dog Breed
Source: PLoS One. 2013 May 31;8(5):e64627. doi: 10.1371/journal.pone.0064627 (PMC3669408; doi:10.1371/journal.pone.0064627)

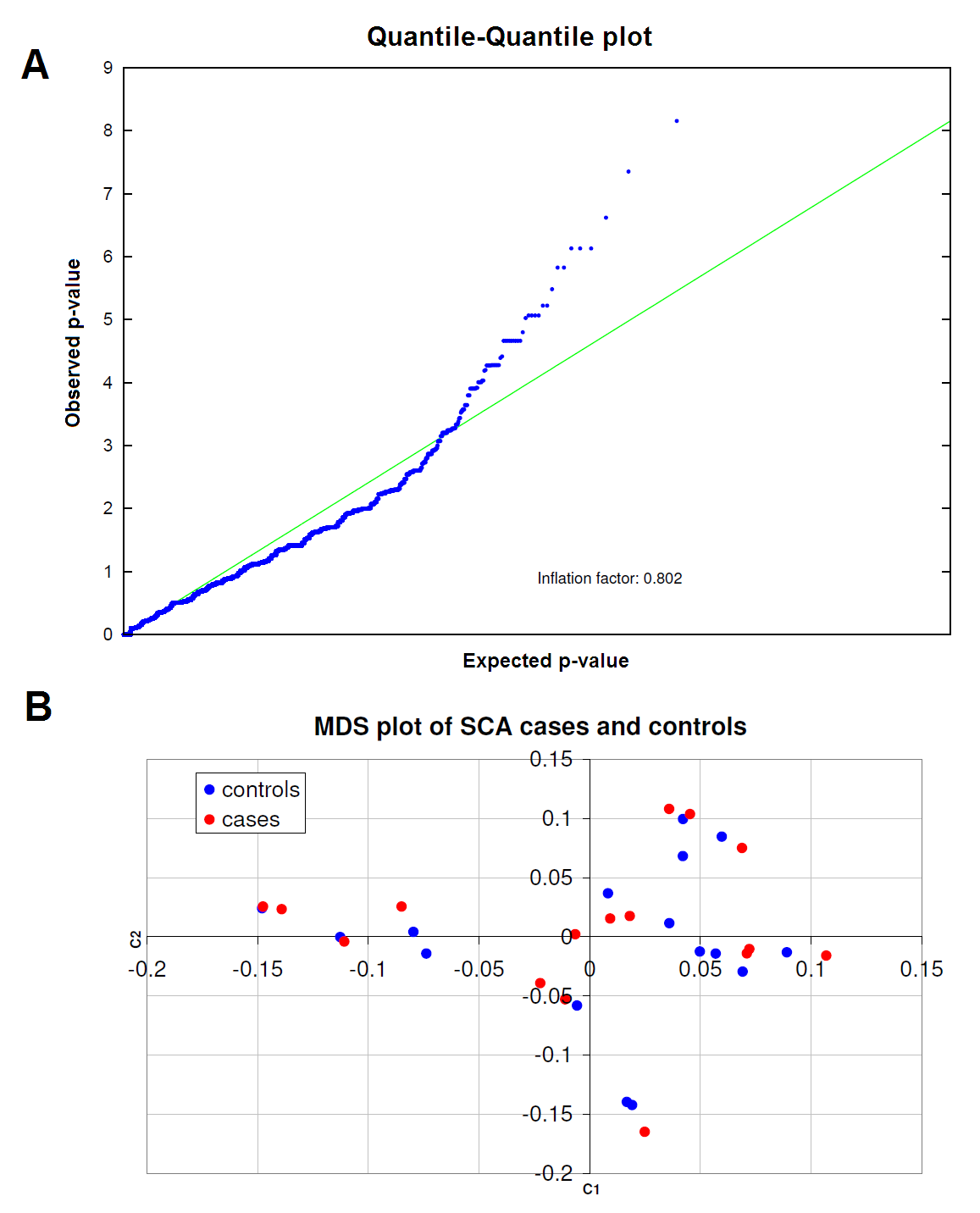

Supplement: Figure S1 — QQ and MDS plots for the GWAS dataset. (TIF) [file pone.0064627.s001.tif]

# PRT SCA association analysis (Fast Mixed Model adjusted)

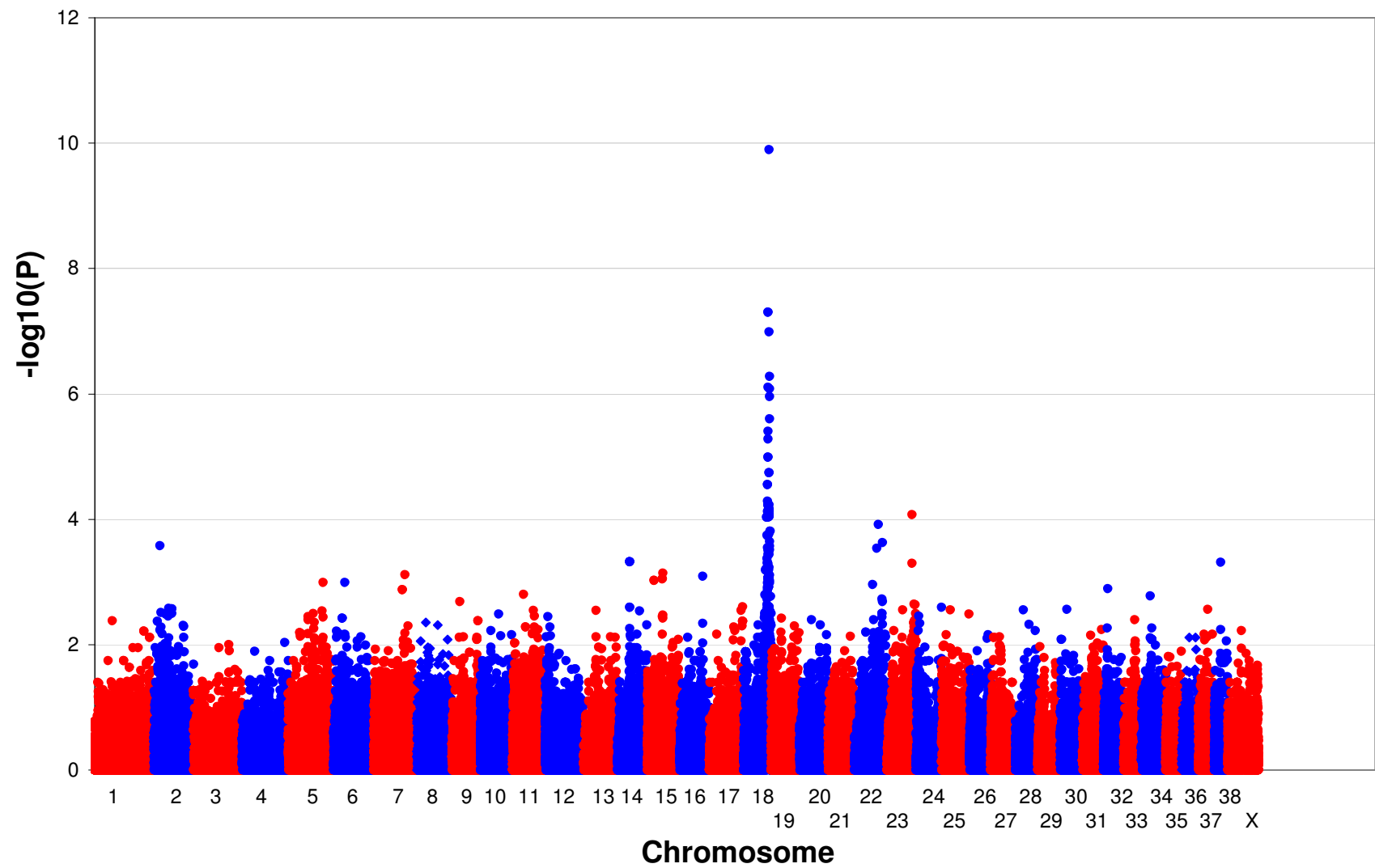

Supplement: Figure S2 — Allelic association analysis plot after correction for population stratification using a mixed model approach. (PDF) [file pone.0064627.s002.pdf]

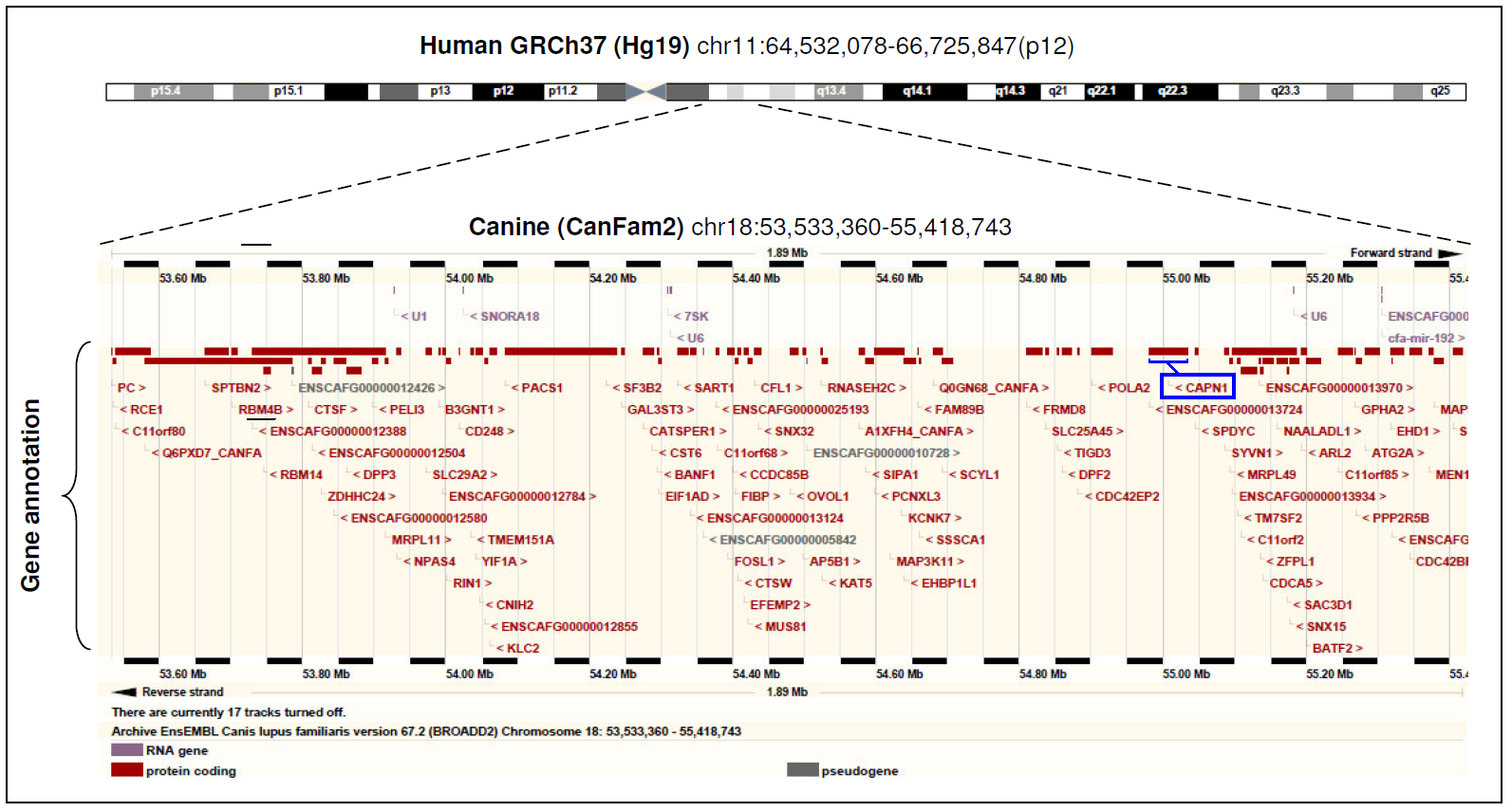

Supplement: Figure S3 — SCA disease-associated interval and human syntenic chromosome. (TIF) [file pone.0064627.s003.tif]
