# Supplementary material for: Missense Mutation in CAPN1 Is Associated with Spinocerebellar Ataxia in the Parson Russell Terrier Dog Breed
Source: PLoS One. 2013 May 31;8(5):e64627. doi: 10.1371/journal.pone.0064627 (PMC3669408; doi:10.1371/journal.pone.0064627)
Supplement: Figure S4 — Genotyping results of the eight outlier cases for 48 SNPs across the disease-associated interval. The outlier cases are not homozygous for the disease-associated haplotype. (PDF) [file pone.0064627.s004.pdf]

				GWAS CASE COHORT																GWAS CONTROL COHORT																OUTLIERS (Individuals with clinical signs of ataxia but not homozygous for the *CAPN1* mutation)							
**Chr**	**SNP name**	**Position**		12118_PRT	12573_PRT	12835_PRT	12854_PRT	14528_PRT	17526_PRT	17545_PRT	17546_PRT	17547_PRT	17933_PRT	18188_PRT	18500_PRT	11445_PRT	11453_PRT	11837_PRT	12015_PRT	11291_PRT	12018_PRT	12837_PRT	12898_PRT	12899_PRT	12947_PRT	17477_PRT	17529_PRT	17553_PRT	18032_PRT	18501_PRT	18502_PRT	11294_PRT	11491_PRT	12125_PRT	12205_PRT	12835_PRT	17760_JRT	18355_JRT	19371_JRT	19936_PRT	21224_PRT	21225_PRT	21150_JRT
18	BICF2P970496	52492443	1.6E-05	G G	G G	G G	G A	G G	G G	G G	G G	G G	G G	G G	G A	G G	G G	G G	G G	G A	G A	G G	A A	A A	G A	A A	A A	G A	G A	G A	G A	A A	G G	G A	G G	G G	G A	G A	A A	G G	G A	G A	G A
18	BICF2P958336	52619327	9.48E-06	A A	A A	A G	A G	A A	A A	A A	A A	A A	A A	A A	A G	A A	A A	A A	A A	A G	A A	A G	G G	A G	A G	G G	G G	G G	A G	A G	A G	G G	A G	A G	A G	A G	A G	A A	G G	A A	A G	A G	G G
18	BICF2P1438978	52840884	7.43E-07	A A	A A	A C	A A	A A	A A	A A	A A	A A	A A	A A	A C	A A	A A	A A	A A	A C	A C	A C	C C	C C	A C	C C	A C	C C	A C	A C	A C	C C	A C	A C	A C	A C	A C	A C	C C	A A	A C	A C	A C
18	BICF2P880099	52985150	5.31E-05	C C	C C	C C	C C	C C	C C	C C	C C	C C	C C	C C	C G	C C	C C	C C	C C	C G	C G	C C	C G	G G	C G	G G	C G	C G	C C	C G	C G	G G	C C	C G	C C	C C	C G	C G	G G	C G	C G	C G	C C
18	BICF2P1127143	53097882	6.31E-05	A A	A A	A G	A A	A A	A A	A A	A A	A A	A A	A A	A A	A A	A A	A A	A A	A G	A G	A A	A G	G G	A G	A A	A A	G G	A G	A G	G G	G G	A A	A A		A G	A A	A A	A G	A A	A G	A G	G G
18	BICF2P448303	53197305	9.93E-05	A A	A A	A G	A A	A A	A A	A A	A A	A A	A A	A A	A G	A A	A A	A A	A A	A A	A G	A A	G G	A G	A A	G G	A G	G G	A G	A G	G G	A G	A G	A G	A A	A G	A G	A G	A G	G G	A A	G G	G G
18	BICF2P129634	53233190	0.000285	A A	A A	A A	A A	A A	A A	A A	A A	A A	A A	A A	A G	A A	A A	A A	A A	A A	A G	A A	A G	A G	A A	G G	A G	A G	A A	A G	A G	A G	A G	A G	A G	A A	A G	A G	G G	G G	A G	G G	A G
18	BICF2P539360	53394191	4.06E-05	G G	G G	G A	G G	G G	G G	G G	G G	G G	G G	G G	G A	G G	G G	G G	G G	G G	G A	G G	A A	G A	G G	A A	G A	A A	G A	G A	A A	G A	G A	G A	G A	G A	G A	G A	A A	A A	G A	A A	G A
18	BICF2P1329203	53468436	0.01041	A A	A A	A G	A A	A A	A A	A A	A A	A A	A A	A A	A G	A A	A A	A A	A A	A A	A A	A A	A G	A G	A A	A A	A A	G G	A G	A A	G G	A G	A A	A G	A G	A G	A A	G G	G G	A G	A G	A G	A G
18	BICF2P76201	53517306	0.08628	G G	G G	G A	G G	G G	G G	G G	G G	G G	G G	G G	G G	G G	G G	G G	G G	G G	G G	G G	G A	G G	G G	G G	G G	G A	G A	G G	A A	G G	G G	G G	G G	G A	G G	G G	G A	G G	G A	G G	G G
18	BICF2P1155995	53644828	2.18E-05	A A	A A	A G	A A	A A	A A	A A	A A	A A	A A	A A	A A	A A	A A	A A	A A	A A	A G	A A	G G	A A	A A	G G	A G	G G	A G	A G	G G	A G	A G	A G	A G	A G	A G	G G	G G	G G	A G	G G	A G
18	BICF2P753571	53696412	5.31E-05	A A	A A	A G	A A	A A	A A	A A	A A	A A	A A	A A	A A	A A	A A	A A	A A	A A	A A	A A	G G	A A	A A	G G	A G	G G	A G	A G	G G	A G	A G	A G	A G	A G	A G	G G	G G	G G	A G	G G	A G
18	BICF2P327014	53729092	2.18E-05	A A	A A	A G	A A	A A	A A	A A	A A	A A	A A	A A	A A	A A	A A	A A	A A	A A	A G	A A	G G	A A	A A	G G	A G	G G	A G	A G	G G	A G	A G	A G	A G	A G	A G	G G	G G	G G	A G	G G	A G
18	BICF2P986209	53791895	2.18E-05	C C	C C	C G	C C	C C	C C	C C	C C	C C	C C	C C	C C	C C	C C	C C	C C	C C	C G	C C	G G	C C	C C	G G	C G	G G	C G	C G	G G	C G	C G	C G	C G	C G	C G	G G	G G	G G	C G	G G	C G
18	BICF2P102870	53848702	0.000632	A A	A A	A C	A A	A A	A A	A A	A A	A A	A A	A A	A A	A A	A A	A A	A A	A A	A C	A A	A C	A A	A A	C C	A C	A C	A C	A A	C C	A A	A C	A C	A C	A C	A A	A C	A A	C C	A A	A C	A C
18	BICF2P1282644	53907984	0.000125	A A	A A	A G	A A	A A	A A	A A	A A	A A	A A	A A	A A	A A	A A	A A	A A	A A	A G	A A	G G	A A	A A	G G	A G	G G	A G	A G	G G	A G	A A	A G	A A	A G	A G	A G	G G	A A	A G	A G	A G
18	BICF2P660897	53932040	0.000125	G G	G G	G A	G G	G G	G G	G G	G G	G G	G G	G G	G G	G G	G G	G G	G G	G G	G A	G G	A A	G G	G G	A A	G A	G A	G A	G A	A A	G G	G A	G A	G A	G A	G A	G A	G G	A A	G G	G A	G A
18	BICF2P1337495	53981168	5.31E-05	A A	A A	A G	A A	A A	A A	A A	A A	A A	A A	A A	A A	A A	A A	A A	A A	A A	A G	A A	G G	A A	A A	G G	A G	G G	A G	A G	G G	A G	A G	A G	A A	A G	A G	G G	A G	G G	A A	G G	A G
18	BICF2P326274	54008916	0.000576	G G	G G	G G	G G	G G	G G	G G	G G	G G	G G	G G	G G	G G	G G	G G	G G	G G	G A	G G	G A	G G	G G	A A	G A	G A	G G	G A	G A	G A	G G	G A	G G	G G	G G	G A	G A	G G	G G	G A	G G
18	BICF2P152937	54062783	2.18E-05	A A	A A	A G	A A	A A	A A	A A	A A	A A	A A	A A	A A	A A	A A	A A	A A	A A	A G	A A	G G	A A	A A	G G	A G	G G	A G	A G	G G	A G	A G	A G	A G	A G	A G	G G	G G	G G	A G	G G	A G
18	BICF2S23645462	54287582	5.31E-05	A A	A A	A G	A A	A A	A A	A A	A A	A A	A A	A A	A A	A A	A A	A A	A A	A A	A G	A A	G G	A A	A A	G G	A G	G G	A G	A G	G G	A G	A G	A G	A A	A G	A G	G G	G G	G G	A G	G G	A G
18	BICF2P680854	54395197	8.66E-06	G G	G G	G A	G G	G G	G G	G G	G G	G G	G G	G G	G G	G G	G G	G G	G G	G G	G A	G G	A A	G G	G G	A A	G A	A A	A A	G A	A A	G A	G A	G A	G A	G A	G A	A A	A A	A A	A A	A A	G A
18	TIGRP2P257487	54419119	0.000576	G G	G G	G G	G G	G G	G G	G G	G G	G G	G G	G G	G G	G G	G G	G G	G G	G G	G A	G G	G A	G G	G G	A A	G A	G G	G A	G A	G G	G G	G A	G A	G A	G G	G G	G G	G G	G A	A A	G A	G G
18	BICF2S23026958	54576931	8.66E-06	G G	G G	G A	G G	G G	G G	G G	G G	G G	G G	G G	G G	G G	G G	G G	G G	G G	G A	G G	A A	G G	G G	A A	G A	A A	A A	G A	A A	G A	G A	G A	G A	G A	G A	A A	A A	A A	A A	A A	G A
18	BICF2P210400	54648370	5.37E-05	A A	A A	A A	A A	A A	A A	A A	A A	A A	A A	A A	A A	A A	A A	A A	A A	A A	A A	A A	G G	A A	A A	G G	A G	A G	G G	A G	G G	A G	A G	A A	A A	A A	A G	A G	- -	A G	G G	G G	A G
18	TIGRP2P257540	54707786	5.37E-05	A A	A A	A A	A A	A A	A A	A A	A A	A A	A A	A A	A A	A A	A A	A A	A A	A A	A A	A A	G G	A A	A A	G G	A G	A G	G G	A G	A G	A G	A G	A A	A G	A A	A G	A G	A G	A G	G G	G G	A G
18	BICF2P1047524	54796248	0.001362	A A	A A	A G	A A	A A	A A	A A	A A	A A	A A	A A	A A	A A	A A	A A	A A	A G	A G	A G	A A	A G	A G	A A	A G	A G	A G	A A	A G	A G	A A	A G	A A	A G	A G	A G	A G	A G	A G	A A	A G
18	BICF2P1238819	54809788	0.000576	A A	A A	A A	A A	A A	A A	A A	A A	A A	A A	A A	A A	A A	A A	A A	A A	A A	A A	A A	C C	A A	A A	C C	A C	A C	A C	A C	A C	A C	A A	A A	A A	A A	A A	A C	C C	A A	A C	A C	A C
18	BICF2P642311	54837807	0.002497	G G	G G	G G	G G	G G	G G	G G	G G	G G	G G	G G	G G	G G	G G	G G	G G	G G	G A	G G	G A	G G	G G	G G	G G	G A	G A	G A	G A	G A	G G	G A	G G	G G	G G	G A	G A	G A	G A	G A	G G
18	TIGRP2P257607	54883940	2.18E-05	G G	G G	G A	G G	G G	G G	G G	G G	G G	G G	G G	G G	G G	G G	G G	G G	G A	G G	G A	G A	G A	G A	G G	G A	A A	A A	G A	A A	A A	G G	G G	G A	G A	G A	G G	G G	G A	G G	G A	G A
18	BICF2P525884	54961775	0.000576	G G	G G	G G	G G	G G	G G	G G	G G	G G	G G	G G	G G	G G	G G	G G	G G	G A	G G	G A	G A	G A	G A	G G	G A	G G	G A	G G	G A	G A	G G	G A	G G	G G	A A	G G	G G	G A	G G	G A	A A
18	BICF2P156099	54981333	1.5E-06	G G	G G	G G	G G	G G	G G	G G	G G	G G	G G	G G	G G	G G	G G	G G	G G	G C	G G	G C	C C	G C	G C	C C	C C	G C	G C	G C	C C	C C	G G	G G	G G	G G	C C	G C	G C	G G	G G	G C	C C
18	BICF2P651617	55033139	7.04E-09	A A	A A	A A	A A	A A	A A	A A	A A	A A	A A	A A	A A	A A	A A	A A	A A	A G	A G	A G	G G	A G	A G	G G	G G	A G	G G	A G	G G	G G	A G	A G	A G	A A	G G	A G	A A	A G	A G	G G	G G
18	BICF2S23514719	55158205	0.000268	G G	G G	G G	G G	G G	G G	G G	G G	G G	G G	G G	G G	G G	G G	G G	G G	G A	G G	G A	G A	G A	G A	G G	G A	G A	G A	G A	G G	A A	G G	G G	G G	G G	G A	G A	G A	G G	G A	G A	G A
18	TIGRP2P257664	55208015	2.18E-05	G G	G G	G A	G G	G G	G G	G G	G G	G G	G G	G G	G G	G G	G G	G G	G G	G G	G A	G A	G A	G A	G G	A A	A A	G A	A A	G A	A A	G G	G A	G A	G G	G A	G A	G A	G A	G A	A A	G A	G A
18	BICF2P363526	55311435	5.37E-05	A A	A A	A A	A A	A A	A A	A A	A A	A A	A A	A A	A A	A A	A A	A A	A A	A A	A G	A A	G G	A A	A G	G G	A G	A G	A A	A G	A G	G G	A A	A A	A G	A A	A G	A G	G G	A A	A G	A G	A G
18	BICF2P647600	55450863	0.08628	G G	G G	G G	G A	G G	G G	G G	G G	G G	G G	G G	G G	G G	G G	G G	G G	G G	G A	G G	G G	G G	G A	G A	G G	G G	G G	G G	G A	G G	G G	G G	G A	G G	G G	G G	G G	G G	G G	G G	G G
18	BICF2P1340374	55491708	0.000122	G G	G G	G G	G G	G G	G G	G G	G G	G G	G G	G G	G G	G G	G G	G G	G G	G G	G A	G A	G A	G G	G G	G A	G A	G A	G A	G A	G A	G A	G A	G A	G G	G G	G G	G A	A A	G G	A A	G A	A A
18	BICF2P1278708	55516531	3.3E-06	A A	A A	A A	A G	A A	A A	A A	A A	A A	A A	A A	A A	A A	A A	A A	A A	A A	A A	A G	G G	A G	A G	G G	G G	A G	A G	A G	A G	G G	A G	A G	A G	A A	G G	A G	G G	A A	G G	A G	G G
18	BICF2S2312188	55597108	0.005885	A A	A A	A A	A G	A A	A A	A A	A A	A A	A A	A A	A A	A A	A A	A A	A A	A A	A A	A G	A G	A A	A G	A G	A A	A G	A A	A G	A A	G G	A A	A A	A G	A A	A A	A G	G G	A A	A A	A G	A A
18	BICF2S23127989	55674540	7.43E-07	A A	A A	A T	A T	A A	A A	A A	A A	A A	A A	A A	A A	A A	A A	A A	A A	A A	A T	A T	A T	A T	A T	T T	T T	T T	T T	A T	T T	T T	A T	A T	A T	A T	T T	T T	A T	A T	T T	T T	A T
18	BICF2P617558	55803431	9.93E-05	A A	A A	A G	A G	A A	A A	A A	A A	A A	A A	A A	A A	A A	A A	A A	A A	A A	A A	A G	A G	A A	A G	G G	A G	G G	G G	A G	A G	G G	A A	A G	A G	A G	A A	G G	G G	A G	A G	G G	A A
18	BICF2P1026445	55860486	0.2002	G G	G G	G A	G G	G A	G G	G A	G A	G A	G A	G G	A A	G G	G G	G G	G G	G G	G G	G G	G G	G A	G G	G G	G G	G A	G A	G G	G G	G G	G G	G G	G G	G A	G G	G A	G G	G A	G G	G A	G G
18	BICF2S2341553	55938478	0.756	G G	G G	G G	G G	G A	G G	G A	G A	G A	G A	G G	A A	G G	G G	G G	G G	G G	G G	G A	G G	G A	G G	G G	G G	G G	A A	G A	G G	G G	G G	G A	G G	G G	G G	G G	G A	G G	G A	G G	A A
18	BICF2P87507	55976410	0.001362	A A	A A	A G	A A	A A	A A	A A	A A	A A	A A	A A	A A	A A	A A	A A	A A	A A	A G	A G	G G	A G	A A	A G	G G	A G	A A	A G	A G	A A	A A	A A	A A	A G	A G	A G	A A	G G	A G	A G	A A
18	BICF2P474131	56051164	0.005366	A A	A A	A G	A A	A G	A A	A G	A G	A G	A G	A A	G G	A A	A A	A A	A A	A A	A G	A G	G G	A G	A A	G G	A G	G G	G G	G G	G G	A G	A A	A G	A G	A G	A G	G G	A G	A G	G G	G G	G G
18	TIGRP2P257922	56056094	0.4118	G G	G G	G A	G G	G A	G G	G A	G A	G A	G A	G G	A A	G G	G G	G G	G G	G G	G G	G A	G A	G A	G G	G A	G G	G A	A A	A A	G G	G G	G G	G A	G A	G A	G A	G A	G G	G A	G A	G A	A A
18	BICF2S22931556	56260105	0.005366	C C	C C	C A	C C	C A	C C	C A	C A	C A	C A	C C	A A	C C	C C	C C	C C	C C	C A	C A	A A	A A	C C	A A	A A	C A	A A	A A	A A	C C	C A	C C	C A	C A	A A	C A	C A	C A	A A	C A	A A
