# Supplementary material for: Missense Mutation in CAPN1 Is Associated with Spinocerebellar Ataxia in the Parson Russell Terrier Dog Breed
Source: PLoS One. 2013 May 31;8(5):e64627. doi: 10.1371/journal.pone.0064627 (PMC3669408; doi:10.1371/journal.pone.0064627)
Supplement: Table S1 — Summary of cases used in the GWAS. (PDF) [file pone.0064627.s005.pdf]

ID	STATUS	Origin	Onset age	Neurologist examined	Video	Case details reviewed by neurologist (LDR)	Clinical signs consistent with case definition?	Matched control ID	Relationship of control to case
11445	Ataxia case	UK	8 months	No	No	Yes	Owner reported case	11491	Half sibling
11453	Ataxia case	UK	unknown	No	Yes	Yes	Yes, video consistent with typical case definition	11294	Half sibling
11837	Ataxia case	UK	10 months	No	No	Yes	Owner reported case	12205	Half sibling
12015	Ataxia case	New Zealand	10-12 months	No	No	Yes	Yes, onset age and vet notes consistent with typical case definition	12125	Half sibling
12118	Ataxia case	Czech Republic	7 months	Yes	Yes	Yes	Yes, referral notes consistent with typical case definition	11291	Mother
12573	Ataxia case	New Zealand	unknown	No	No	Yes	Owner reported case	12018	Mother
12835	Ataxia case	UK	<6 months	No	No	Yes	No, hypermetric front limb gait and horizontal head twitch and onset age not consistent with typical case definition	12837	Father
12854	Ataxia case	UK	unknown	No	No	Yes	Owner reported case	12947	Sibling
14528	Ataxia case	UK	12 months	Yes	Yes	Yes	Yes, examined by two independent veterinary neurologists	-	-
17526	Ataxia case	USA	7-8 months	No	No	Yes	Owner reported case	12899	Half sibling
17545	Ataxia case	Belgium	unknown	No	No	Yes	Owner reported case	17477	Unrelated at grandparent level
17546	Ataxia case	Belgium	unknown	No	No	Yes	Owner reported case	17529	Unrelated at grandparent level
17547	Ataxia case	Belgium	unknown	No	No	Yes	Owner reported case	17553	Unrelated at grandparent level
17933	Ataxia case	USA	8 months	No	No	Yes	Owner reported case	18032	Unrelated at grandparent level
18188	Ataxia case	UK	unknown	No	No	Yes	Owner reported case	18502	Sibling
18500	Ataxia case	Holland	7 months	Yes	Yes	Yes	Yes, video consistent with typical case definition	18501 &12899	Mother & Half sibling
