# Supplementary material for: Missense Mutation in CAPN1 Is Associated with Spinocerebellar Ataxia in the Parson Russell Terrier Dog Breed
Source: PLoS One. 2013 May 31;8(5):e64627. doi: 10.1371/journal.pone.0064627 (PMC3669408; doi:10.1371/journal.pone.0064627)
Supplement: Table S2 — Summary of the 142 SNPs and indels that segregated with disease status. (PDF) [file pone.0064627.s006.pdf]

Chromosome	Base position	Type	Consequence
chr18	54147546	SNP	G_INTRONIC
chr18	54806784	SNP	T_DOWNSTREAM
chr18	54834860	SNP	T_UPSTREAM
chr18	54834925	SNP	T_UPSTREAM
chr18	54835424	SNP	A_UPSTREAM
chr18	54835743	SNP	A_UPSTREAM
chr18	54835906	SNP	G_UPSTREAM
chr18	54836122	SNP	A_UPSTREAM
chr18	54837638	SNP	T_INTRONIC
chr18	54840102	SNP	C_INTRONIC
chr18	54841610	SNP	T_DOWNSTREAM
chr18	54841654	SNP	A_DOWNSTREAM
chr18	54844842	SNP	A_DOWNSTREAM
chr18	54845696	INDEL	INS_DOWNSTREAM
chr18	54845982	SNP	G_DOWNSTREAM
chr18	54858698	SNP	A_UPSTREAM
chr18	54869070	INDEL	DEL_INTRONIC
chr18	54872648	SNP	A_UPSTREAM
chr18	54872962	SNP	T_UPSTREAM
chr18	54873168	SNP	A_UPSTREAM
chr18	54873206	INDEL	INS_UPSTREAM
chr18	54919810	SNP	A_INTRONIC
chr18	54934495	SNP	T_INTERGENIC
chr18	54938409	SNP	A_INTERGENIC
chr18	54941771	SNP	T_INTERGENIC
chr18	54942354	SNP	T_INTERGENIC
chr18	54945643	SNP	A_INTERGENIC
chr18	54946289	SNP	T_INTERGENIC
chr18	54946549	INDEL	INS_INTERGENIC
chr18	54949116	SNP	A_INTERGENIC
chr18	54950071	SNP	A_INTERGENIC
chr18	54979508	INDEL	INS_DOWNSTREAM
chr18	54982957	SNP	T_INTRONIC
chr18	54987373	SNP	A_INTRONIC
chr18	54988675	SNP	A_INTRONIC
chr18	55002285	SNP	A_INTRONIC
chr18	55015088	SNP	T_INTRONIC
chr18	55032013	SNP	T_INTRONIC
chr18	55033641	SNP	A_INTRONIC
chr18	55034306	SNP	T_NON_SYNONYMOUS_CODING CAPN1 C115Y
chr18	55051929	SNP	T_UPSTREAM
chr18	55068865	SNP	T_INTERGENIC
chr18	55072232	SNP	T_INTERGENIC
chr18	55083317	SNP	C_UPSTREAM
chr18	55084753	SNP	A_UPSTREAM
chr18	55104243	SNP	C_DOWNSTREAM
chr18	55105267	SNP	A_INTRONIC
chr18	55107297	SNP	A_INTRONIC
chr18	55107498	INDEL	DEL_INTRONIC
chr18	55107873	SNP	C_INTRONIC
chr18	55109826	SNP	A_INTRONIC
chr18	55110148	INDEL	DEL_INTRONIC
chr18	55113209	SNP	G_INTRONIC
chr18	55113424	SNP	C_INTRONIC
chr18	55115671	SNP	G_INTRONIC
chr18	55115691	SNP	G_INTRONIC
chr18	55120823	SNP	G_INTRONIC
chr18	55121762	INDEL	DEL_INTRONIC

chr18	55129178	INDEL	DEL_INTRONIC
chr18	55129602	SNP	G_INTRONIC
chr18	55130944	SNP	T_NON_SYNONYMOUS_CODING VPS51 E24K
chr18	55131116	SNP	C_INTRONIC
chr18	55137241	SNP	A_INTRONIC
chr18	55138179	SNP	T_INTRONIC
chr18	55146289	SNP	A_INTRONIC
chr18	55146383	SNP	G_INTRONIC
chr18	55146756	SNP	A_INTRONIC
chr18	55149036	SNP	G_INTRONIC
chr18	55152325	SNP	A_INTRONIC
chr18	55152649	SNP	T_INTRONIC
chr18	55153753	SNP	A_INTRONIC
chr18	55155557	SNP	G_INTRONIC
chr18	55157953	SNP	A_INTRONIC
chr18	55159180	SNP	A_INTRONIC
chr18	55173974	SNP	T_INTRONIC
chr18	55175535	INDEL	INS_INTRONIC
chr18	55188478	SNP	T_DOWNSTREAM
chr18	55188984	SNP	C_DOWNSTREAM
chr18	55192173	SNP	T_UPSTREAM
chr18	55194978	SNP	A_INTRONIC
chr18	55195382	SNP	A_INTRONIC
chr18	55200159	SNP	C_INTRONIC
chr18	55222654	SNP	T_DOWNSTREAM
chr18	55249754	INDEL	DEL_INTRONIC
chr18	55352472	SNP	A_NON_SYNONYMOUS_CODING CDC42BPG G689R
chr18	55354056	SNP	A_INTRONIC
chr18	55371811	SNP	A_UPSTREAM
chr18	55372281	SNP	G_SYNONYMOUS_CODING
chr18	55377582	SNP	T_DOWNSTREAM
chr18	55382018	SNP	A_DOWNSTREAM
chr18	55382921	SNP	A_INTRONIC
chr18	55383138	SNP	T_INTRONIC
chr18	55384340	SNP	A_INTRONIC
chr18	55385447	SNP	A_INTRONIC
chr18	55401520	SNP	G_UPSTREAM
chr18	55405494	SNP	T_INTRONIC
chr18	55405973	SNP	C_INTRONIC
chr18	55451852	SNP	T_DOWNSTREAM
chr18	55511405	SNP	A_INTRONIC
chr18	55523280	SNP	T_INTRONIC
chr18	55524712	SNP	T_INTRONIC
chr18	55588876	SNP	C_INTERGENIC
chr18	55607893	SNP	A_INTRONIC
chr18	55618004	SNP	C_INTERGENIC
chr18	55634143	SNP	T_INTERGENIC
chr18	55634376	SNP	C_INTERGENIC
chr18	55635088	INDEL	DEL_INTERGENIC
chr18	55638891	SNP	A_INTERGENIC
chr18	55640395	SNP	G_INTERGENIC
chr18	55641250	SNP	T_INTERGENIC
chr18	55641937	INDEL	INS_INTERGENIC
chr18	55642330	SNP	A_INTERGENIC
chr18	55644502	SNP	T_INTERGENIC
chr18	55644705	SNP	G_INTERGENIC
chr18	55645554	SNP	T_INTERGENIC
chr18	55645725	SNP	A_INTERGENIC
chr18	55647128	SNP	G_INTERGENIC

chr18	55647279	SNP	T_INTERGENIC
chr18	55647608	SNP	C_INTERGENIC
chr18	55655406	SNP	A_INTERGENIC
chr18	55655666	SNP	A_INTERGENIC
chr18	55655809	SNP	A_INTERGENIC
chr18	55655946	SNP	G_INTERGENIC
chr18	55655954	SNP	A_INTERGENIC
chr18	55655983	SNP	G_INTERGENIC
chr18	55656250	SNP	T_INTERGENIC
chr18	55658118	SNP	G_INTERGENIC
chr18	55660886	SNP	T_INTERGENIC
chr18	55662353	SNP	C_INTERGENIC
chr18	55663080	SNP	A_INTERGENIC
chr18	55663100	SNP	T_INTERGENIC
chr18	55667091	SNP	A_INTERGENIC
chr18	55667274	SNP	T_INTERGENIC
chr18	55667372	SNP	A_INTERGENIC
chr18	55667619	SNP	C_INTERGENIC
chr18	55670711	SNP	A_INTERGENIC
chr18	55682394	SNP	T_INTERGENIC
chr18	55698803	SNP	T_INTERGENIC
chr18	55701874	SNP	A_INTERGENIC
chr18	55724206	SNP	A_INTERGENIC
chr18	55736847	SNP	C_INTRONIC
chr18	55747955	SNP	A_UPSTREAM
