# Supplementary material for: Missense Mutation in CAPN1 Is Associated with Spinocerebellar Ataxia in the Parson Russell Terrier Dog Breed
Source: PLoS One. 2013 May 31;8(5):e64627. doi: 10.1371/journal.pone.0064627 (PMC3669408; doi:10.1371/journal.pone.0064627)
Supplement: Table S3 — List of primer names and sequences used in the study. (DOC) [file pone.0064627.s007.doc]

**Table S1**

***SPTBN2* sequencing primers**

| Exon | Forward primer | Sequence | Reverse Primer | Sequence | Size |
| --- | --- | --- | --- | --- | --- |
| 1 | SPTBN2_1_F | GACGGAATTCTCCTGGTTGA | SPTBN2_1_R | GTGGGTGCCCCCTATAATG | 452 |
| (2)+3 | SPTBN2_(2)+3_F | ACTTTTAGCTCCCAGCTTTGC | SPTBN2_(2)+3_R | TACTCTTGGCACTGGAATTGG | 644 |
| 4 | SPTBN2_4_F | GACCTCACTTCTGGGGTCTTC | SPTBN2_4_R | GGCTTGGGCAGATTAAGATTC | 538 |
| 5 | SPTBN2_5_F | GCTTTGGCTCTGTGAACTTTG | SPTBN2_5_R | TGGATATCACAGCAACCACAA | 484 |
| 6+7 | SPTBN2_6+7_F | TGACTTCTGACCCATTCTGCT | SPTBN2_6+7_R | CCAGCTCTTGTCTGGATGTTC | 666 |
| 8 | SPTBN2_8_F | GGGCACCACACTACATAAAGG | SPTBN2_8_R | AAAGGAAACCCCTTACCCATT | 425 |
| 9+10 | SPTBN2_9+10_F | ATTCGGGGTGAGTGATTCAAG | SPTBN2_9+10_R | GATTGCTTCTGGCACTTTGAG | 693 |
| 11 | SPTBN2_11_F | ATTTTCTGGCTTTTGCACTGA | SPTBN2_11_R | GGGGCGTGTTGTACAAGATAA | 446 |
| 12 | SPTBN2_12_F | TCCAGTCCTCACCTAGAAGCA | SPTBN2_12_R | AACCCTTTCTCCTGTTCAAGC | 552 |
| 13 | SPTBN2_13_F | GAGGCTTGAACAGGAGAAAGG | SPTBN2_13_R | GCCCTGTTTGCTCTGTGTATC | 616 |
| 14 | SPTBN2_14_F | GACAGAACTGGGGTGTCACTG | SPTBN2_14_R | CCCTAATGCTGTCACCTACCA | 526 |
| 15.1 | SPTBN2_15.1_F | TGGTAGGTGACAGCATTAGGG | SPTBN2_15.1_R | TCTGCCTGGAACTGGTAGAGA | 581 |
| 15.2 | SPTBN2_15.2_F | CTTGAAGCTTACGCTGGAACA | SPTBN2_15.2_R | TTGTAGCAAGAGCAGGCAGTT | 685 |
| 16 | SPTBN2_16_F | CTCCCCACTTGACCCATAGAT | SPTBN2_16_R | GCTCATTCTCCCTTGACTCCT | 518 |
| 17.1 | SPTBN2_17.1_F | AGGAGTCAAGGGAGAATGAGC | SPTBN2_17.1_R | ATCTAAGCTGCGCAGGAAGTC | 576 |
| 17.2 | SPTBN2_17.2_F | GGCTTGGAGAGGTACAAGCTG | SPTBN2_17.2_R | TATGTTCTCCCAGTCCACCAG | 600 |
| 18 | SPTBN2_18_F | GTTTGGGCTGTTAAGGTCCTC | SPTBN2_18_R | GCACTATCCAGCTGCTTCATC | 499 |
| 19 | SPTBN2_19_F | GTGGTCAGAATGGGACTAAGC | SPTBN2_19_R | GCATATGCAGTGAAGCATAAGG | 460 |
| 20 | SPTBN2_20_F | GCTCTTGGTTCCTCCATGTC | SPTBN2_20_R | GGAGGTTTGGCACTAAAGACC | 513 |
| 21 | SPTBN2_21_F | GGTCTCTGTGGCTTCTTTGTG | SPTBN2_21_R | GACGTGGCCTTGAGAAATACC | 658 |
| 22 | SPTBN2_22_F | ACTGAGGTAGGCCATGAGGAT | SPTBN2_22_R | ACTTTCAGTGCCAAAGGGAAG | 503 |
| 23 | SPTBN2_23_F | TTCCCTTTGGCACTGAAAGTA | SPTBN2_23_R | CCTAAGGTTGTTAGGCCTTCG | 499 |
| 24 | SPTBN2_24_F | GTGGGTTTCCATTGTAGCAGA | SPTBN2_24_R | TGCTTCTTTACCTCTGCTTGG | 574 |
| 25 | SPTBN2_25_F | TCCAAGAGATGTGGAAACACC | SPTBN2_25_R | TGTACCAAAGGCTCCACAGTC | 471 |
| 26 | SPTBN2_26_F | AGCCAAGACATGATTGACCAC | SPTBN2_26_R | TGACCTCCTTTCTGCACAGTT | 627 |
| 27 | SPTBN2_27_F | TGCTCTGCAGAAGAAACCTGT | SPTBN2_27_R | GCATCCTCCTAGCATTTCTGA | 617 |
| 28 | SPTBN2_28_F | CTCCTGCTTCTCTGATGGATG | SPTBN2_28_R | GGCCTCTATCTCTGCCTTGAT | 529 |
| 29 | SPTBN2_29_F | TACTGGACACCACGGACAAGT | SPTBN2_29_R | GGCAGAGACGTGAGTTAGCAC | 578 |
| 30 | SPTBN2_30_F | TCCAACTCTTCTCCAGAACCA | SPTBN2_30_R | CAGAGAAATGGCAGGTCAGAG | 461 |
| 31 | SPTBN2_31_F | GGCAGTAGCTTCCTCCATGTC | SPTBN2_31_R | TCCTGAAGCTGCAGTGACAAT | 450 |
| 32+33 | SPTBN2_32+33_F | TACCCATTGTCACTGCAGCTT | SPTBN2_32+33_R | GCTGCTCTGCTCAAGTCTCTG | 657 |
| 34 | SPTBN2_34_F | AGGAGGAAGAACGGAGGAAAC | SPTBN2_34_R | GGGAAAGTGAGGAATGGTGTC | 624 |
| 35 | SPTBN2_35_F | CCCAGAGGTGTGGGTGTTAAT | SPTBN2_35_R | CCAGAGGACCCTCCTCCTTAC | 543 |
| 36 | SPTBN2_36_F | CGAGCTCAGAAACAGGAACAG | SPTBN2_36_R | TGGATGTTGTCAGTGGTCAGG | 501 |
| 37 | SPTBN2_37_F | ACAGGAAGGGTTTGGGAATCT | SPTBN2_37_R | GAAGCTGGAAAGCCTACGAGA | 477 |
| 38 | SPTBN2_38_F | CCAGTGAGGTGAAGTAGTCCAG | SPTBN2_38_R | AAAGAAGTTGGGAGATGGGAGT | 607 |

**Allelic discrimination primers and probes**

| Assay name | Forward Primer Seq. | Reverse Primer Seq. | Reporter 1 Sequence  (MGB, 5’VIC, 3’ NFQ) | Reporter 2 Sequence  (MGB, 5’FAM, 3’ NFQ) |
| --- | --- | --- | --- | --- |
| CAPN1 | GGGTGAGGGAGGCAATGG | GCAGAGCAGGCCTGATATGG | AGAAGCCAACAGTCCC | AGAAGCCAATAGTCCC |
| VPS51 | CCTCCGAGAGACCGTAGTAAATC | GGTCTGGAACTGGGGACTCTC | AGGCGGAGGCCC | AGGCGAAGGCCC |

**Genotyping-by-sequencing primers**

| BICF2P448303F | TGCCTGGTGCTGATACCTG | 126 |
| --- | --- | --- |
| BICF2P448303R | CTTATAGGCCATGGAGGATGG |  |
| BICF2P539360F | GCTCTGTGAGTGATGGCTCTT | 136 |
| BICF2P539360R | TAGACTGGCCTTCGTCTGAAA |  |
| BICF2P1155995F | CAACCTTGGTGCTACTCCAGA | 111 |
| BICF2P1155995R | TACCTGTGAGCAATTGTGCTG |  |
| BICF2P753571F | AAGAAGTTCACCATGGGTCAG | 112 |
| BICF2P753571R | TGGTCAAGAGAGTCATGAGTAAAGA |  |
| BICF2P327014F | AAGAGTGATGGGTCCACCTTT | 120 |
| BICF2P327014R | ACTTTCCTTGCATTTGGCTTT |  |
| BICF2P986209F | CACATGGTAGCAAAGGAGAAA | 121 |
| BICF2P986209R | TCCAGGAACTTTAAAGGCTGTT |  |
| BICF2P1337495F | GCTCGGAGGTAAGGTACAACC | 140 |
| BICF2P1337495R | GGGACCTGTCTCCTTTGTTTC |  |
| BICF2P152937F | GGTATAGGGCTCCCAAGCTG | 110 |
| BICF2P152937R | ATGCTAGGATGGGCCTAGTTC |  |
| BICF2S23645462F | GTGGAGGGTGTTGCTTCTGT | 110 |
| BICF2S23645462R | AAACCACAGTACACCCTTGGA |  |
| BICF2P680854F | CCGTATTGTATTTCACAGAATCG | 144 |
| BICF2P680854R | TGGCATTTCATTGATGCTAAG |  |
| BICF2S23026958F | GGGTGGGTGTGTAGCTGTTAT | 119 |
| BICF2S23026958R | TGACAGTTGAGATGCACAGATG |  |
| BICF2P210400F | GGTTTCCAGCTCTGTGGTTATC | 134 |
| BICF2P210400R | CTGCATGGAGCCAGACAC |  |
| TIGRP2P257540F | TTTGTGAGTGTGACATGATGGA | 122 |
| TIGRP2P257540R | TTTGGCAGGTGCTTAAGAGAA |  |
| TIGRP2P257607F | GCTCTGGGACTGAGTCTAACCA | 112 |
| TIGRP2P257607R | ACCAGGCACAGAACAGAGTCC |  |
| BICF2P156099F | CCTGGAAAGACCTCCTTTGAC | 126 |
| BICF2P156099R | TGGTAGAGACACACATCGTGAA |  |
| BICF2P651617F | TCCCTCATTTGAGGTGTATGG | 158 |
| BICF2P651617R | AACCTGTGAATGCAGGAACC |  |
| TIGRP2P257664F | CATAGCAGGTTTCCTCCACAG | 115 |
| TIGRP2P257664R | CACTCCTCTGGATCCAACTCA |  |
| BICF2P363526F | CAGGTGCTTCCCATCAAATAA | 117 |
| BICF2P363526R | GAGCAGGTTGTTTAAAGCCACTA |  |
| BICF2P1278708F | TTGTGTACAGAGAGGTCACATGG | 114 |
| BICF2P1278708R | CCATGCTCCCACTTGTATTGT |  |
| BICF2S23127989F | AACATAGTCAAGATGGTGCATGTAT | 115 |
| BICF2S23127989R | GGAAGTTGGCCCTGAGTAAAG |  |
| BICF2P617558F | CATTCACAGGAGTTGGGAGAG | 115 |
| BICF2P617558R | TTTCAACCGCAAGATCAGTTT |  |
| BICF2P87507F | CATGAAACATGATGAAATCTGCT | 122 |
| BICF2P87507R | CTGCGGTCTCCACAGTCTC |  |
| TIGRP2P257922F | CCAGCTCACACCCATTCC | 115 |
| TIGRP2P257922R | ACGTGCCATAGGCTGTTAGG |  |
| BICF2S22931556F | TTTGCCACAGTTTGTCAGCTA | 118 |
| BICF2S22931556R | CTGAGCGTCACGGGTCTTA |  |
| BICF2P970496_F | AAAGCCCTCGTTTCCACAG | 107 |
| BICF2P970496_R | TCTTAGCTGGAGGTGCCTTG |  |
| BICF2P958336_F | AGCCATCCTCCACCCTGTAG | 110 |
| BICF2P958336_R | AATGCCAGACAGAGCAGCTT |  |
| BICF2P1438978_F | AGGGAGTGGGTTTCATCCTC | 119 |
| BICF2P1438978_R | ATCAGCTGGTGGGTGCTATC |  |
| BICF2P880099_F | GGCTGACTTTCCAGAATCCA | 108 |
| BICF2P880099_R | CCCTTCTCACACTGGTCTGTC |  |
| BICF2P1127143_F | TTGTCTACCAGCCGTTTGC | 128 |
| BICF2P1127143_R | CATGGAAGGCAAGAATAGCC |  |
| BICF2P129634_F | TTCATGTCTGGGTAGGAGATCA | 114 |
| BICF2P129634_R | TCCCTCAGTATGGGTAAGTGTTC |  |
| BICF2P102870_F | ACACTGCTGTACGCTTCAGG | 113 |
| BICF2P102870_R | TGGAGGAAAGACCAGGAATG |  |
| BICF2P1282644_F | ACCAACTGCTTTGCCAACTG | 123 |
| BICF2P1282644_R | GTGGCCTCTCAGGGAGATTC |  |
| BICF2P660897_F | ATGGGCTTCAAATCTGTTGG | 127 |
| BICF2P660897_R | ATTGGAGGCTTCTTGCTCAT |  |
| BICF2P326274_F | CCACCGTCACAGAGCATTTA | 106 |
| BICF2P326274_R | TTCTGCGCACAAACATGAGT |  |
| TIGRP2P257487_F | TGGCCAGGTACCTTGTTTATG | 109 |
| TIGRP2P257487_R | TTAAGAGCCAGGGTTTCTGC |  |
| BICF2P1047524_F | AGACCATGTGGCCTCCATT | 108 |
| BICF2P1047524_R | TTCTGGCAGAGAGTTGGTCA |  |
| BICF2P1238819_F | TTTGATGCTGGGTCAGCTCT | 116 |
| BICF2P1238819_R | TATGGAGGCTCACCTTCAGC |  |
| BICF2P525884_F | GGCTGGCTTTCAGAAGACAC | 111 |
| BICF2P525884_R | GTGATGATGATGCCATGAGG |  |
| BICF2S23514719_F | GACCCATGAGCGAAGTAGGA | 116 |
| BICF2S23514719_R | ATCTTGGCTAGGTGGGTGGT |  |
| BICF2P647600_F | TGCTGAGAACAGTCTGAGGAAC | 127 |
| BICF2P647600_R | CACTTCTGGAAGGCTCGATG |  |
| BICF2P1340374_F | GAGACACTGCCGATGAGACA | 113 |
| BICF2P1340374_R | TTTAAGCCGTCAAGGAACGTA |  |
| BICF2P1026445_F | GCGGACACACAGCTGAAAG | 106 |
| BICF2P1026445_R | GGGCTGCTCCCTTATCTCAC |  |
| BICF2S2341553_F | ACTGCCACTGCCCTTGTGT | 111 |
| BICF2S2341553_R | AAGACCCAGGATGAGGGAGT |  |
| BICF2P474131_F | CTGGCATTCTGTCACCATGA | 109 |
| BICF2P474131_R | ACAGACACCCTGTGCGTGAC |  |
| BICF2P1329203_F | AAAGATAAGCCTGCCCAAAT | 124 |
| BICF2P1329203_R | GAAGACAACGCCCTCTCACT |  |
| BICF2P76201_F | GTTTCCTGGCCCTCTAGCTC | 128 |
| BICF2P76201_R | CATGTCCCACTTCCTTGGAG |  |
| BICF2P642311_F | GCCACCATGTCTTTCTACAGC | 103 |
| BICF2P642311_R | TTGCTGTAGACTCCAAACAGGA |  |
| BICF2S2312188_F | TTGGGTAGAGTTCAGGCTTGT | 129 |
| BICF2S2312188_R | ACACCACTGCAACCCTCTTC |  |
